# Molecular Characterization and Antimicrobial Resistance of Pathogenic *Escherichia coli* Strains in Children from Wolaita Sodo, Southern Ethiopia

**DOI:** 10.1155/2022/9166209

**Published:** 2022-07-06

**Authors:** Amanuel Wolde, Yosef Deneke, Tesfaye Sisay, Mesfin Mathewos

**Affiliations:** ^1^College of Agriculture, Department of Veterinary Science, Jinka University, Jinka, Ethiopia; ^2^School of Agriculture and Veterinary Medicine, Jimma University, Jimma, Ethiopia; ^3^Institute of Biotechnology, Addis Ababa University, Addis Ababa, Ethiopia; ^4^School of Veterinary Medicine, Wolaita Sodo University, Wolaita Sodo, Ethiopia

## Abstract

**Introduction:**

Pathogenic *Escherichia coli* strains cause diarrheal infection in children due to their virulence factors. A nonanalytical observational study followed by a purposive sampling technique was conducted from October 2017 to June 2018, to determine the antimicrobial susceptibility patterns and molecularly detect pathogenic *Escherichia coli* strains in under-five children at Wolaita Sodo town using molecular and the Kirby-Bauer disc diffusion method.

**Result:**

In the current investigation, out of 110 stool samples, *Escherichia coli* was isolated in 68 (61.8%) (95% CI: 52.1–70.9%). Out of 68 *Escherichia coli* isolates, 61.9% of *Escherichia coli* isolates were resistant, 9.4% were intermediately resistant, and 28.7% were susceptible. Among the antimicrobial agents, 91.2% of *Escherichia coli* isolates were highly sensitive to ciprofloxacin followed by norfloxacin (86.8%). Virulence genes were detected in 55.9% (38/68) (95% CI 52.1%–70.9%) of isolates. The following genes were detected: *stx1* (6 (8.8%)), *stx2* (4 (5.9%)), *eaeA* (5 (7.4%)), *eaat* (14 (20.5%)), and *St* (4 (5.9%)). Out of 68 *Escherichia coli* isolates, 43 (63.2%) isolates had shown multidrug resistance patterns. Furthermore, 11.8% of *Escherichia coli* isolates had shown resistance to eight different drugs. The multidrug resistance index value of Diarrheagenic *Escherichia coli* pathotypes was greater than or equal to 0.4, which indicates the high risk of resistance.

**Conclusion:**

This study demonstrated important pathogenic *Escherichia coli* strains and multidrug resistance in isolates containing virulence genes. Wise use of antimicrobials and improving the hygienic practices amongst parents of children reduce its occurrence. Therefore, appropriate usage of antimicrobial agents should also be highly practiced in hospitals.

## 1. Introduction

In developing countries, children, particularly those under the age of five years, are vulnerable to diarrhea, which is one of the leading causes of mortality and morbidity [1, 2], resulting in the deaths of nearly 2.6 million people each year, the majority of whom are African children [3]. In 2010, it was anticipated that over 1.7 billion episodes of diarrhea occurred worldwide, with 700,000 of those resulting in death in children under the age of five years [4, 5].

Children's diarrhea is a frequent ailment in most impoverished countries, including Ethiopia. Consumption of contaminated foods, such as undercooked meat and unpasteurized milk, close contact with reservoir animals, caregivers' lack of hand-washing with soap, lack of proper breastfeeding for children until the age of six months, and poor health infrastructure have all been linked to bacterial pathogens that cause diarrhea [6]. According to a global review, infections (sepsis, pneumonia, tetanus, and diarrhea) and preterm delivery resulted in newborn fatalities, which were responsible for 4 million neonatal deaths [7]. However, the early beginning of breastfeeding (or human milk feeding) and exclusive nursing can avoid or decrease the negative consequences of infections and preterm delivery [8].

Diarrhea syndromes can be caused by single or many etiologic agents such as bacterial, viral, or parasite diseases [9]. Enteric bacterial pathogens, and their products, are the major causes of acute diarrhea, including diarrheagenic *Escherichia coli (*DEC), nontyphoidal *Salmonella* (NTS), *Shigella* spp., and *Vibrio* cholera, among others [10, 11].

Among the causal agents of diarrhea in humans and animals, diarrheagenic *Escherichia coli* (DEC) is the most frequent of the numerous enteropathogenic organisms, especially in poor countries and in diarrhea-related mortality in children under the age of five [12]. These bacteria are genetically the most versatile and are the source of many plasmid and phage-mediated genes. Even though its members are typically nonpathogens that are a part of the normal microflora of the intestinal tract of humans and animals, certain subsets of this bacterial species have acquired genes that enable them to cause intestinal or extraintestinal disease [13, 14].

Diarrheagenic *Escherichia coli* are identified and differentiated from *Escherichia coli* of the intestinal flora by the presence of specific virulence markers [15]. This bacterium, which is classified under the family *Enterobacteriaceae*, is Gram-negative, rod-shaped, flagellated, motile, oxidase-negative, and facultatively anaerobic. It produces septicemia and diarrhea in a wide range of hosts including humans and animals [16].

Pathogenic *Escherichia coli* strains are divided into intestinal pathogens causing diarrhea and extraintestinal *Escherichia coli* (ExPEC) causing a variety of infections in both humans and animals including urinary tract infections, meningitis, and septicemia [17]. Pathogenic *Escherichia coli* causing diarrhea in children are also further divided into two types: enteropathogenic *Escherichia coli* and uropathogenic *Escherichia coli.* Uropathogenic *Escherichia coli* causes 90% of the urinary tract infection. Based on their specific virulence factors, phenotypic traits, and mechanisms by which they cause disease, DEC strains are divided into six pathotypes: enteropathogenic *Escherichia coli* (EPEC), enterotoxigenic *Escherichia coli* (ETEC), Vero toxin-producing/Shiga toxin-producing *Escherichia coli* (VTEC/STEC), which include its well-known subgroup enterohemorrhagic *Escherichia coli* (EHEC), enteroinvasive *Escherichia coli* (EIEC), enteroaggregative *Escherichia coli* (EAEC), and diffusely adherent *Escherichia coli* (DAEC) [18].

Improper usage of antimicrobial agents is one of the main reasons for the pathogenic bacteria to develop antibiotic resistance to the commonly used antimicrobial agents [19]. According to [20], the prevalence of antibiotic resistance among *Escherichia coli* isolates from patients with acute diarrhea in Egypt was 68.2%, 57.2%, and 24.2% for ampicillin, trimethoprim-sulfamethoxazole, and ampicillin-sulbactam, respectively. In contrast, studies in Vietnam found that 86.4%, 77.2%, and 19.1% of *Escherichia coli* isolates were resistant to resistant to ampicillin, chloramphenicol, and trimethoprim-sulfamethoxazole, respectively [21]. The widespread use of these antibiotics has resulted in an increased prevalence of resistance to these antibiotics by diarrheagenic bacteria, thereby raising concern in children, especially in developing countries [22].

*Escherichia coli* also represents a major reservoir of resistance genes that may be responsible for treatment failures in both human and veterinary medicine. An increasing number of resistance genes have been identified in *Escherichia coli* isolates during the last decades, and many of these resistance genes were acquired by horizontal gene transfer [23] or clonal transfer of resistant strains via direct contact, indirect contact, and consumption of fecally contaminated food [24].

The identification of DEC is done based on the presence of different chromosomal and/or plasmid-encoded virulence genes that are absent in commensal *Escherichia coli* via molecular methods [25]. Although different virulence groups of DEC can also be detected by culture, biochemical reactions, serotyping, and other phenotypic assays [26], polymerase chain reaction (PCR) has proved to be more sensitive and specific than most conventional techniques and has been used to identify DEC in several studies [27]. Antimicrobial resistance and virulence types in *Escherichia coli* have been studied in a variety of countries [28–34]. Limited studies on antimicrobial susceptibility patterns and molecular detection of pathogenic *Escherichia coli* strains in diarrheic children at Wolaita Sodo town initiated this research work [21]. Therefore, the objective of the current study was to determine antimicrobial susceptibility patterns and molecularly detect pathogenic *Escherichia coli* strains in diarrheic children at Wolaita Sodo town.

## 2. Materials and Methods

### 2.1. Study Area

**Figure 1:**
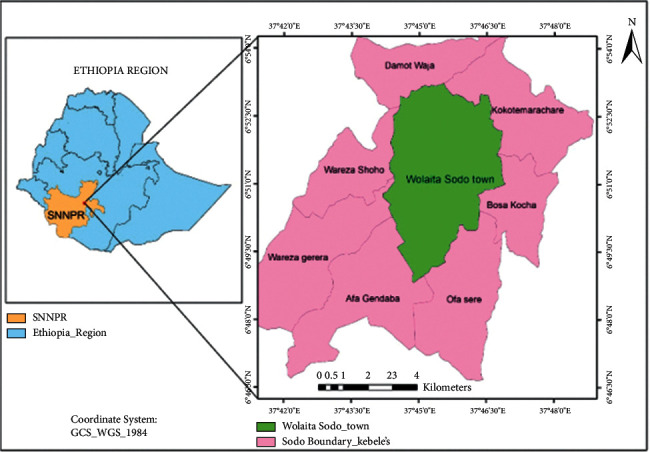
Map of the study area.

The current research work was carried out in the Sodo Christian Hospital from October 2017 to June 2018. Wolaita Sodo is a town in the Southern nation nationalities and people of the republic (SNNPR) situated 383 kilometers from Addis Ababa. The region is bordered on the north by Damot gale woreda, on the south by Humbo woreda, on the east by Damot Wilde woreda, and on the west by Damot Sore woreda (Figure 1). It has an annual rainfall of 100–1200 mm and a temperature of 25–35°C. Its height ranges from 1650 to 2980 meters above sea level. The area is Woina-Dega (mid-altitude) at an elevation below 1600 feet, and the livestock population in the Wolaita Sodo zone includes 128,919 cattle, 29191 sheep, and 4606 equines, and 55278 poultry [35]. According to the results of the May 2007 housing and population census, Wolaita Sodo town has a population of 102,922, with 54,315 men and 48,617 females, and a 5.3% annual population growth rate. This makes Wolaita Sodo the second most populous city in South Region after Hawassa. Mixed farming, which includes the development of grain crops, cash crops, especially coffee, and animal production, is the most important economic activity [36].

### 2.2. Study Population

All under-five children with diarrhea who visited Sodo Christian hospital in Wolaita Sodo town were included in the study as described by Black et al. [35]. Diarrheic children under the age of five who attended Sodo Christian Hospital with diarrhea and whose caregivers were willing to participate in the study were included in the study's sample population.

### 2.3. Study Design

A nonanalytical observational study starting from October 2017 to June 2018 was conducted in the Sodo Christian Hospital found in Wolaita Sodo town. The Sodo Christian hospital was chosen purposefully, based on the availability of clinical cases (diarrheic youngsters) and the willingness of the children's parents. The pediatrician provided information about the children's health. Children with normal stool consistency and/or indications of dehydration, sunken eye, diarrhea, and weakness were categorized as healthy, whereas ill children with abnormal stool consistency and/or signs of dehydration, sunken eye, diarrhea, and weakness were classified as diarrhea. The kind, features, and color of diarrhea were also documented.

#### 2.3.1. Sample Size Determination

The selection of participants from Sodo Christian hospital and stool samples from children was based on a nonprobability purposive sampling technique. During sampling, the availability of clinical cases (diarrheic children) and the willingness of the parents of the children were the main factors considered to determine the total number of stool samples. Accordingly, 110 stool samples were collected from children that have diarrhea.

#### 2.3.2. Sample Collection

The sample collection procedure was applied by medical laboratory technicians having the stool laboratory request of the pediatricians for diarrheic children under-five years of age who visit Sodo Christian hospital. The stool samples were collected by researchers, and it was collected from the diaper of the children. The collected stool samples were labeled properly and aseptically transported to the Wolaita Sodo regional veterinary laboratory in an icebox containing ice packs and then immediately processed for bacterial isolation.

#### 2.3.3. Isolation and Identification of *Escherichia coli*

*Escherichia coli* was isolated and identified based on the standard procedures described by Quinn et al. [37] and the techniques recommended by the ISO [38]. After immediately arriving at the laboratory and/or overnight storage in the refrigerator at 4°C and thawing at room temperature, the samples were manually homogenized by using a vortex mixer for about 40 seconds. When there is a small number of samples in a sterile flask, 25 g of fecal samples were mixed into 225 mL of sterile buffered peptone water (Himedia, India) in a 1 : 9 ratio. The preenriched samples were homogenized in the flask for two minutes before being incubated aerobically for 24 hours at 37°C. All the study's media were prepared according to the manufacturer's instructions. The sample dilution of preenriched broth was inoculated aseptically onto sterile MacConkey agar (Himedia, India) and incubated at 37°C for 24 hours. The presence of growth on MacConkey agar was employed as the major criterion for moving on with *Escherichia coli* isolation and identification. A few representative *Escherichia coli* colonies were subcultured on Eosin methylene blue (EMB) agar medium (Himedia, India) to confirm their identity as *Escherichia coli.* The green metallic sheen appearance of *Escherichia coli* colonies on EMB was used to identify their features. For biochemical testing, all isolated colonies were maintained on a nutrient agar slant (Oxoid, England). Gram staining was used to evaluate the morphology and purity of all isolates [39], and they were subjected to the standard biochemical tests: Indole test, Methyl red (MR), Voges Proskauer (VP) test, Citrate test, and triple sugar iron. The isolates that exhibited an IMViC pattern (++−−) were presumed to be *Escherichia coli* isolates. All the presumed *Escherichia coli* isolates were subcultured on a nutrient agar slant (Oxoid, England) for antimicrobial testing.

#### 2.3.4. Antimicrobial Susceptibility Testing

Antimicrobial susceptibility testing was performed according to the “Kirby and Bauer” method on Mueller-Hinton agar medium [40], following the standard agar disk diffusion method [41] using antimicrobial disks of commonly used drugs such as Ampicillin, Chloramphenicol, Ciprofloxacin, Clindamycin, Neomycin, Norfloxacillin, Oxytetracycline Streptomycin, Sulphonamides, Tetracycline, and Trimethoprim. Following this, the diameter of the inhibition zone formed around each disc was measured using a calibrated ruler. The results were classified as sensitive, intermediate, and resistant according to the standardized table supplied by the manufacturer of the discs [41]. Also, the multidrug resistance index (MDRI) of individual isolates was calculated by dividing the number of antimicrobial agents to which the isolate was resistant by the total number of drugs to which the isolate was exposed. Values lower than 0.2 are considered low risk, while a value higher than 0.2 is considered high risk [42].

#### 2.3.5. Molecular Characterization of *Escherichia coli* Isolates

*(1) DNA Extraction*. Bacterial DNA extraction was performed using the boiling method. A Loopful colony was taken from each medium for inoculation of *Escherichia coli* isolates in nutrient broth at 37°C overnight. Exactly 1.5 ml of the young nutrient broth culture was harvested and spanned by centrifugation at 13,000 rpm for 10 minutes in a sterilized Eppendorf tube. The supernatant was discarded; then, 50 *µ*l of nuclease-free water was added to the pellet and boiled to lyse in a water bath at 95°C for 10 minutes. It was then centrifuged again as before, and an aliquot of the supernatant was transferred to another autoclaved Eppendorf tube as the test DNA template and stored at −20°c for later use in PCR experiments. Three *µ*l of the extracted DNA was used directly as a template for PCR amplification [43].

*(2) Polymerase Chain Reaction*. After extraction of the target DNA, all *Escherichia coli* isolates were subjected to PCR to detect virulence genes. The optimized PCR protocol was carried out with a PCR mix of a 25 *μ*l mixture containing 16 *μ*l double distilled water, 2.5 *μ*l 10x PCR buffer with MgCl2, 1 *μ*l dNTP, 1 *μ*l forward primer, 1 *μ*l reverse primer, 0.5 *μ*l *Taq* DNA polymerase (5 U/*µ*l) (Himedia India), and 3 *μ*l of template DNA. Negative control was performed by adding 3 *μ*l of sterile nuclease-free water; a positive control was performed by adding 3 *μ*l of a known DNA sample. For those strains where positive control is not available in the laboratory, pooled target DNA samples were used as a positive control. According to optimized PCR conditions, the amplification of the reaction mixtures was performed in a PCR machine, Eppendorf thermal-cycler, Biometra, Geotting, Germany. The primer sequences and optimized PCR protocol used in this work and their features are indicated in Table 1.

*(3) Agarose Gel Electrophoresis*. Amplified PCR products were separated by agarose gel electrophoresis at 120 volts for 30 minutes in 1.5% agarose containing Ethidium Bromide in 1x in TBE buffer using a marker 100 bp DNA ladder (Himedia; India and Promega, Madison, Wisconsin, USA). Loading dye was used to load PCR products in each well in a gel. The products were then visualized by a UV transilluminator [50] and photographed under ultraviolet light using a digital Polaroid camera.

### 2.4. Data Analysis

All of the data were subject to contingency table analysis, and Pearson's Chi-square test was used to determine the statistical significance of the relationships and or associations between the virulence genes and corresponding pathogenic *Escherichia coli* strains. Throughout the investigation, a significance level of *p* < 0.05 at 95% confidence intervals was employed. All data from the laboratory tests were coded, filtered, and recorded in Microsoft Excel spreadsheet 2007 (Microsoft Corporation) before being analyzed with SPSS version 20.0 software (SPSS INC. Chicago, IL).

### 2.5. Ethical Considerations

This study received ethical approval from the Jimma University of Research Ethics and Review Committee. Before collecting samples, the parents or guardians of the patients were asked for verbal agreement to collect samples from their children while adhering to stringent sanitary guidelines. All of the studies were done following the Helsinki Declaration. The best practices for human care were followed, and the patients' parents or guardians were told of the study's objective and that the Jimma University of Research Ethics and Review Committee authorized the oral informed consent process.

## 3. Results

### 3.1. Pathogenic *Escherichia coli* Strains Identified

**Figure 2:**
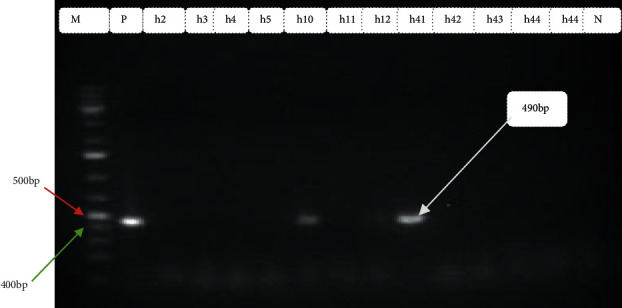
Amplification of *eae* gene in *Escherichia coli* isolates from diarrheic children: M Marker (100 bp DNA ladder) P positive control, Lane No. h10 and h41 indicate positive samples, Lane No. h2-h5, and h43-h44 indicates negative samples and N negative control.

**Figure 3:**
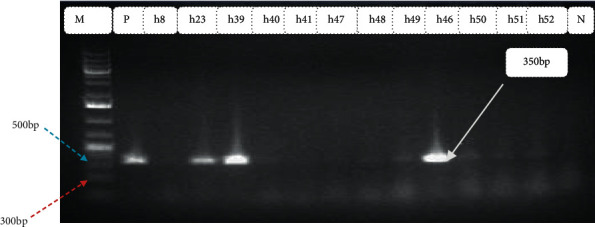
Amplification of stx1 gene in *Escherichia coli* isolates from diarrheic children: M Marker (100 bp DNA ladder) P positive control, Lane No. h23, h39, and h49 indicate positive samples, Lane No. h8, h40, h41, h47, h48, h49, h51, and h52 indicates negative samples and N negative control.

**Figure 4:**
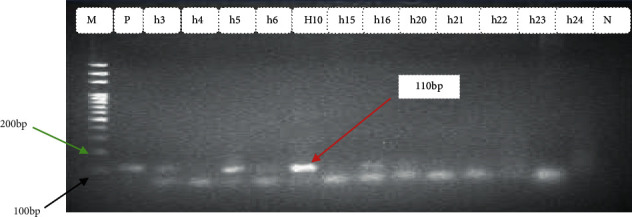
Amplification of *stx2* gene in *Escherichia coli* isolates from diarrheic children: M Marker (100 bp DNA ladder) P positive control, Lane No. h5, and h10 indicates positive samples, Lane No. h3, h4, h6, h15, h16, and h20–h25 indicates negative samples and N negative control.

**Figure 5:**
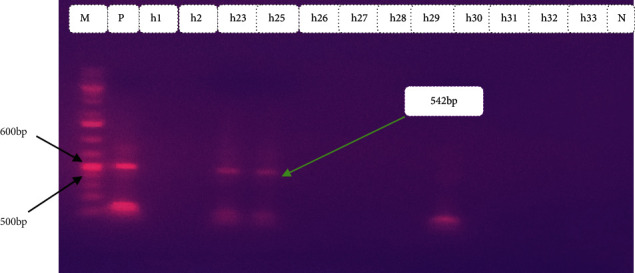
Amplification of *dae* gene in *Escherichia coli* isolates from diarrheic children: M marker (100 bp DNA ladder) P positive control, lane no. h23, and h25 indicates positive samples, lane no. h1, h2, and h26–h33 indicates negative samples and N negative control.

In the current investigation, out of 110 stool samples, *Escherichia coli was* isolated in 68 (61.8%) (95% CI: 52.1–70.9%). Among those *Escherichia coli isolates*, 38 (55.8%) were detected as harboring one or more virulence genes (Figures 2–5). Out of these, 35 (92.1%) isolates harbored one virulence gene, and the rest 3 (7.9%) isolates harbored more than one virulence gene. According to the occurrence of virulence genes, the 38 *Escherichia coli* isolates were classified as follows: 14 EAEC (eaat gene), 10 STEC (6 harbored stx1 gene and 4 harbored the stx2 gene), 2 EHEC (1 harbored stx 2+ eaeA genes, 1 harbored as stx 1+ eaeA genes), 5 atypical EPEC (eaeA gene), 4 ETEC (st gene), and 2 DAEC (data gene) (Table 2). All isolates were checked for the presence of the *bfp* gene, but none of them contained this gene. Also, neither EHEC nor STEC harbored the *hlyA* gene. All virulence genes detected were significantly associated (*p* < 0.05) with diarrheal infection due to *Escherichia coli* in children.

Typical gel pictures for the specific virulence genes investigated are indicated in Figures 2–5.

### 3.2. Antimicrobial Susceptibility Patterns of *Escherichia coli* Isolates

#### 3.2.1. Mono-Drug Resistance

The antimicrobial susceptibility patterns of 68 *Escherichia coli* isolates were shown in Table 3. Accordingly, the highest sensitivity to ciprofloxacin (91.2%) followed by norfloxacin (86.8%) was observed in *Escherichia coli* isolates. Chloramphenicol, clindamycin, neomycin, streptomycin, and norfloxacin did not show intermediate resistance, while the other tested drugs showed intermediate resistance in two or more of the tested isolates. The highest resistance was observed to neomycin (97.1%) followed by streptomycin (95.6%).

#### 3.2.2. Multidrug Resistance

Multidrug resistance patterns of the *Escherichia coli isolates* are shown in Table 4. *Of the 68 Escherichia coli* isolates, 43 (63.2%) were resistant to two or more (up to ten) antimicrobials, respectively. Eight (11.8%) of *Escherichia coli isolates* had shown resistance to seven different drugs, while 7 (10.3%) of *Escherichia coli* isolates had shown resistance to eight drugs.

The STEC, EPEC, EAEC, EHEC, DAEC, and St (ETEC) strains in diarrheic children were highly resistant to neomycin and ampicillin 38 (100%) and clindamycin 36 (94.7%). Moderately high resistance was detected towards norfloxacin 18% (47.4) and chloramphenicol 24 (63.2%). The DEC pathotypes were least resistant to ciprofloxacin, 2 (5.3%) (Table 5).

Thirty-eight (100%) of the *Escherichia coli* strains showed multidrug resistance, and 3 (7.9%) strains showed resistance to at least four drugs and at most to ten drugs (Table 6).

Four (10.5%) and three (7.9%) strains were found to have the highest (0.9) and lowest (0.4) MDR index values, respectively, out of a total of 38 stool samples from diarrheic children (Table 7). In diarrheic children, a calculated MDR index value greater than or equal to 0.3 and 0.4 suggests a high chance of resistance.

## 4. Discussion

Diarrhea is a multifactorial illness caused by a complex interaction between the body, its environment, diet, and infectious pathogens [51]. In the current study, *Escherichia coli* isolates were found in 68 (61.8%) of the 110 stool samples, which were higher compared with the findings of [52–54], and [55] who reported an isolation rate of 31% in Austria, 48.3% in Bahir Dar, 22.6% in Mozambique, and 2.3% in Nepal from diarrheic under-five years children. It was, however, lower than the 86.5% reported by [56] in Kenya and the 88% reported by [57] in Nigeria. Different sociodemographic factors, sample size variance, and the season when the research was conducted could all contribute to the difference in isolation rate [58].

Antimicrobial resistance levels of Ampicillin, chloramphenicol, tetracycline, and streptomycin for *Escherichia coli* isolates were revealed to be 48 (70.6%), 38 (55.9%), 44 (64.7%), and 65 (95.6%), respectively. The resistance levels of *Escherichia coli* isolates to ampicillin were approximately similar to those [21, 27, 53, 54, 59], and [54] who reported a resistance level of 86% in Vietnam, 73% in Mexico, 72% in Mozambique, and 84% in Kenya, 89% and 93.3% in Iran, but lower resistant levels were previously reported by [60] who revealed that 55.6% of *Escherichia coli* isolates were resistant to ampicillin. The extensive and indiscriminate use of antibiotics, as well as the creation of beta-lactamase enzymes, may be contributing to *Escherichia coli* resistance to ampicillin as already described by [61]. Contrary to this finding, the moderate resistance levels of *Escherichia coli* isolates for chloramphenicol by 27%, tetracycline by 16%, and streptomycin by 9% were previously reported by [27, 62].

The resistance of *Escherichia coli* isolates to tetracycline in our study was consistent with reports from Mexico [27] and Iran [63], which found 85% and 83% resistance, respectively. This could be due to indiscriminate antibiotic use, which was supported by [64]. The current study's high susceptibility of *Escherichia coli* isolates to ciprofloxacin (91.2%) contradicted previous findings by [27, 65], which found 97.2% and 100% susceptibility of *Escherichia coli* isolates to ciprofloxacin, respectively.

In this study, 63.2% of *Escherichia coli* isolates were found to be multidrug resistant. Multidrug resistance in *Escherichia coli* isolates was highest at seven drugs, followed by eight drugs, which was similar to the findings of [53, 56], who found multidrug resistance in *Escherichia coli* isolates.

In the current study, from 68 *Escherichia coli* isolates, 55.9% of DEC with five distinct pathotypes was detected, which was responsible for 30–40% of acute diarrhea episodes in children as indicated by [66]. All virulence genes detected in each pathogenic *Escherichia coli* strain were significantly associated with diarrheal infection. This study revealed a higher frequency of DEC as compared to [15, 67, 68], and [69] who reported 23% and 18.4% by [70] in Nigeria. However, it was slightly agreed with [66] in Iran, [71] in India, and [72] in Sweden, who reported 30.4%, 52%, and 48% DEC pathotypes, respectively.

In the present study, EAEC was the most isolated *Escherichia coli* pathotype with a prevalence of 14 20.5% followed by EHEC (14.7%), EPEC (11.8%), ST (5.9%), and DAEC (2.9%). Similarly, in the report [69], EAEC was the most commonly isolated category, followed by EPEC and ETEC. These three pathotypes of diarrheagenic *Escherichia coli* were also detected most frequently in children with acute diarrhea in Brazil by [67], in Tanzania by [15], and in Lybia by [73]. Moreover, [66, 67, 74, 75], and [69] also reported 10.7%, 14%, 3.86%, 8%, and 12.2% of EAEC pathotype, respectively. The high frequency of EAEC described in our study, along with the high resistance to antibiotics, supports the need for follow-up epidemiological studies, pathogenesis, and its role in the different forms of diarrhea [76]. An increasing number of studies support the association of EAEC with diarrhea in populations in developing countries [77]. It has also been shown that EAEC is a heterogeneous group of *Escherichia coli* and that not all strains are capable of causing diarrhea [3].

STEC (14.7%) was the second most prevalent strain, which was higher than the previous report [74] in Peru recorded a prevalence of 1%. Also, in another study conducted by [66], 7(1.6%) were positive for *stx1* only, and 6 (1.3%) were positive for *stx2* only.

Atypical EPEC (eaeA+ and bfp) (11.76%) was the third most prevalent strain. In this study, all isolates were identified as atypical EPEC. However, typical EPEC were more prevalent than atypical EPEC in children less than 2 years old in the study conducted in Mexico by [27], and epidemiological studies in several countries showed that atypical EPEC strains have become a more frequent cause of diarrhea than typical EPEC [78]. These observations have been linked to the duration of the diarrheal disease [21, 79].

However, 39 (17.7%) and 45 (40.5%) *Escherichia coli* isolates in Thailand by [80] and in Iran by [81] were reported, respectively, are possessing the *eaeA* gene. Also, [82] found that all EPEC isolates harbored the *eaeA* gene. Ochoa et al. [74] reported a 7% prevalence of EPEC in stool samples and such pathotype was also isolated with a prevalence of 19.3% in Kenya by [75] and 5.1% in Mexico by [69]. Atypical and typical EPEC were detected in 9.4% and only in one sample, respectively, in Brazil, which was reported by [67].

On the other hand, a lower percentage of *eaeA* gene distribution among EPEC was reported in Japan by [83] who showed that all EPEC strains isolated from children with diarrhea did not react with *eaeA-*specific primer. Also, in a report of [22], from 21 EPEC strains, 13 had only the *eaeA* gene, only one strain harbored the *bfp* gene, and 5 strains were positive for *eae* and *bfp*. None of the 77 serological identified EPEC strains contained the *eaeA* gene in a study conducted by [84]. Atypical EPEC is an emergent enteric pathogen that has only recently begun to attract the attention of investigators [85].

A prior study in Brazil and other countries found that enterotoxigenic *Escherichia coli* (ETEC) was the most usually isolated pathotype in children under the age of five who had diarrhea [3, 86]. All *Escherichia coli* isolates were tested for the presence of heat-labile and heat-stable toxins in this study, but none of the isolates tested positive for heat-labile toxin (Lt), and only 4 (5.8%) tested positive for heat-stable toxin (St). This result was lower than the previous reports of [3, 87], and [66] who recorded 21.6%, 20.5%, and 14.4% of ETEC, respectively. Furthermore, Vila [22] isolated 12.7% of ETEC strains with 33 strains (75%) that produced the heat-stable toxin (St), 6 strains (14%) synthesized the heat-labile toxin (Lt), and 5 strains (11%) produced both toxins. On the other hand, a low isolation rate of Enterotoxigenic *Escherichia coli* was reported in Peru by [74], in South India by [71], Kenya by [75], Mexico by [69], and Brazil by [67] who reported an isolation rate of 4%, 4.1%, 7.25%, 4.3%, and 3.7%, respectively.

EHEC was another *Escherichia coli* strain that was found in 3 (4.4%) of the 68 isolates and which was found in higher [3, 67, 71, 87, 88], and [66] who identified a frequency of 1.6%, 0.6%, 2.1%, 1.3%, 2.0%, and 3.8% from various regions of the world. In contrast, [69] in Mexico and [75] in Kenya found EHEC with isolation rates of 0.3% and 0.97%, respectively. In another study [66], 1 (0.2%) out of 17 EHEC strains was tested positive for stx1/eaeA, whereas 3 (0.7%) were tested positive for stx2/eaeA. On the contrary, Canizalez-ROman [69] were not found EHEC strains on his study.

The current study's high frequency of DAEC strains 2 (2.9%) differed from the work of [69] in Mexico and [71] in South India, which reported 1.4% and 0.5% of DAEC strains, respectively, but was lower than the report described by [74] in Peru, which indicated an isolation rate of 4%. Furthermore, DAEC strains were the second most often isolated pathotype linked with diarrhea in Brazilian children aged 2 to 5 years who lived in low socioeconomic areas [89]. DAEC strains have been recovered often from adults with diarrhea in Brazil, according to Mansan-Almeida [90].

EPEC strains were tested for the presence of the virulence EPEC gene in *Escherichia coli* isolates, but none of them carried the gene, which contradicted the findings of [22] in Tanzania, who showed that 1 EPEC strain had just the bfp gene, and 5 strains were eae and bfp positive. The hlyA gene was also not found in EHEC or STEC.

In this study, MDR was found in all pathogenic *Escherichia coli* strains (38 (100%)). Other authors [69] in Northwestern Mexico, [27, 68] in Iran, and [60] in Nigeria, on the other hand, found MDR of diarrheagenic *Escherichia coli* of over 80%, 62%, 67%, and 25.9%, respectively. However, in their studies, [91] in India and [74] in Peruvian reported that only 75% and 63% of pathogenic *Escherichia coli* strains were found to be MDR, respectively. Multidrug resistance in pathogenic *Escherichia coli* strains could be mostly owing to acquired antimicrobial resistance phenotypes, which are most commonly acquired through conjugative transfer of plasmid genes [92].

Plasmid genes may contain class I integrons, which are mobile DNA elements that have a role in the spread of bacterial MDR, particularly among Gram-negative enteric bacteria. Five (100%) atypical EPEC, four (100%) St (ETEC), and fourteen (100%) EAEC resisted ampicillin in diarrheic youngsters in this investigation. Similarly, Roy [93] found that ampicillin was resistant to EPEC in 96% of cases, ETEC in 73% of cases, and EAEC in 100% of cases. Isolated pathogenic *Escherichia coli* strains in Thailand also exhibited strong resistance to ampicillin [94]. According to [69], EPEC and DAEC strains had the highest rates of resistance to ampicillin and chloramphenicol. STEC, EPEC, EAEC, and EHEC are all extremely sensitive to ciprofloxacin in this investigation. In contrast to these findings, in a study conducted by [93], ciprofloxacin resistance was shown to be 39% in EPEC, 36% in ETEC, and 38% in EAEC. Despite the findings of this investigation, EPEC and DAEC strains showed the highest rates of ciprofloxacin resistance, as previously reported by [69]. 70% of DEC strains were reported to be resistant to ampicillin in research conducted on diarrheic children in Mexico [27], but most strains were found to be sensitive to ciprofloxacin. According to [95], the high prescription rate of ciprofloxacin in the study area as a treatment for enteric infections caused by Gram-negative bacteria is the source of varied rates of resistance to this antibiotic.

The resistance patterns among the DEC groups in our investigation were significantly varied. DAEC and EAEC were likely to have higher resistance levels than STEC, which was consistent with the findings of [69], who found the EPEC and DAEC categories to be the most resistant. This is due to their frequent exposure to antimicrobials, as well as the fact that they produce chronic diarrhea and/or are frequently carried asymptomatically. As a result, long-term use of human hosts raises the risk of antimicrobial exposure and/or the acquisition of resistant genes from the resident flora. As previously noted by [96], this study could represent an association of resistance genes with plasmid-associated virulence genes, such as adherence factors present in DAEC (i.e., Dr. Adhesins) and EAEC (i.e., AAF or aggregative adherence fimbria). Multiple antibiotic resistance has been linked to a range of mobile genetic elements, including plasmids, transposons, and gene cassettes in integrins [97] as well as changes in the *Escherichia coli* multiple antibiotic resistance operon (*mar*) [98].

The high degree of resistance is most likely the outcome of selection pressure caused by the uncontrolled and inappropriate use of these Antimicrobials in hospitals and across the country. Antimicrobial resistance in pathogenic bacteria, as well as the formation of resistant strains in flora bacteria, could result from improper usage of antimicrobial drugs. Historically, much of the focus has been on pathogenic bacteria; however, according to [99], the significance of commensal organisms as a reservoir or vehicle for transferring resistance genes to more dangerous, pathogenic bacteria has lately been proposed.

## 5. Conclusion

The overall high proportion of *Escherichia coli* isolates and their significant pathogenic strains indicates the wide distribution of the infection in children in the study area. *Escherichia coli* isolate showed susceptibility mostly to ciprofloxacin and norfloxacin; therefore, they are considered the best choice of treatment. A higher proportion of *eaat* gene-positive *Escherichia coli* strains was detected in children. Multiple drug resistance was mostly observed in pathogenic *Escherichia coli* strains harboring one or more virulence genes. The calculated MDR index value for all *Escherichia coli* pathotypes indicates the high risk of resistance. Antibiotics dosage and frequency should be used inappropriate methods to minimize drug resistance. Further research using whole-genome DNA sequencing and Phylogenetic tree analysis in diarrheic children should be done for the detection of variant genes, and emerging multidrug resistance encoding genes for all molecularly detected *Escherichia coli* strains in this study.

## Figures and Tables

**Table 1:** Primer sequences specific for each virulence gene, their amplicon size, and optimized PCR protocol.

Target gene	Primer name	Sequence 5′ to 3′	Amplicon size (bp)	PCR amplification						*Escherichia coli* strain detected	References
				Initial denaturation C/min	Denaturation °C/sec.	Annealing °C/sec	Extension °C/sec	Final extension °C/min	No. of cycle		
*eaeA*	EAE1	F: AAACAGGTGAAACTGTTGCC	490	95/3	95/40	55/60	72/60	72/10	35	EPEC/EHEC	[44]
	EAE2	R: CTCTGCAGATTAACCTCTGC									
*Stx 1*	EVS1	F: ATCAGTCGTCACTCACTGGT	110	95/3	95/40	57/40	72/30	72/8	30	STEC/EHEC	[45]
	EVC2	R: CTGCTGTCACAGTGACAAA									
*Stx 2*	EVT1	F: CAACACTGGATGATCTCAG	350	95/3	95/40	57/40	72/30	72/8	30	STEC/EHEC	
	EVT2	R: CCCCCTCAACTGCTAATA									
*Bfp*	bfpA1	F: TTCTTGGTGCTTGCGTGTCTTTT	324	95/3	95/40	57/40	72/30	72/8	30	Typical EPEC	[44]
	bfpA2	R: TTTTGTTTGTTGTATCTTTGTAA									
*HlyA*	hlyA-1	F: GGT GCA GCA GAA AAA GTT GTA G	190	95/3	95/40	45/60	72/60	72/10	30	EHEC	[46]
	hlyA-2	R: TCT CGC CTG ATA GTG TTT GGT a									
*aatA*	aatA1	F: GGT GCA GAA CAC GAT GCA	590	95/3	95/40	45/60	72/60	72/10	30	EAEC	[44]
	aatA2	R: CCA CGT CTT GTG CTA CGT									
*Lt*	LT-1	F: TCTCTATGTGCATACGGAGC	696	95/3	95/40	53/60	72/30	72/8	30	ETEC	[47]
	LT-2	R: CCATACTGATTGCCGCAAT									
*St*	ST-1	F: TGCTAAACCAGTAGAGTCTTCAAAA	294	95/3	95/40	52/60	72/60	72/10	35	ETEC	[48]
	ST-2	R:GCAGGATTACAACACAATTCACAGCAG									
*Dae*	*dae*E 1	F: GAA CGT TGG TTA ATG TGG GGT AA	542	95/3	95/40	45/60	72/60	72/10	37	DAEC	[49]
	*dae*E 2	R: TAT TCA CCG GTC GGT TAT CAG									

EPEC = enteropathogenic *Escherichia coli*; ETEC = enterotoxigenic *Escherichia coli;* STEC = shiga toxin-producing *Escherichia coli*; EHEC = enterohaemorrhagic *Escherichia coli*; EAEC = enteroaggregative *Escherichia coli*; DAEC = diffusely adherent *Escherichia coli*; EAE = effacing and attaching, Stx = shiga toxin, bp = Base pair.

**Table 2:** Frequency of virulence genes and corresponding pathogenic *Escherichia coli* strains in diarrheic children.

Virulence genes detected	Frequency of virulence genes among *Escherichia coli isolates* (*N* = 68)	Pathogenic *Escherichia coli* strains	*χ* ^2^	*p*-value (95% CI)
stx 1	6 (8.8%)	STEC	15.1	0.02 (7.3–25.4%)
stx 2	4 (5.9%)	STEC		
stx 2+ eaeA	1 (1.5%)	EHEC	6.8	0.041 (0.9–12.4%)
stx 1+ eaeA	2 (2.9%)	EHEC		
eaeA^+^ only	5 (7.4%)	Atypical EPEC	7.4	0.003 (2.4–16.3%)
St	4 (5.9%)	ETEC	10	0.002 (1.6–14.4%)
Data	2 (2.9%)	DAEC	9.4	0.01 (0.4–10.2%)
Eaat	14 (20.5%)	EAEC	13.9	0.01 (17.7–32.1%)
Total	38 (55.9%)			

(*χ*^2^)-chie square; CI-confidence interval; *p*-probability.

**Table 3:** Antimicrobial susceptibility patterns in *Escherichia coli* isolates.

Antimicrobial agents	Susceptible	Intermediate	Resistant
Ampicillin	8 (11.8)	12 (17.6)	48 (70.6)
Chloramphenicol	30 (44.1)	—	38 (55.9
Ciprofloxacin	62 (91.2)	2 (2.9)	4 (5.9)
Clindamycin	5 (7.4)	—	63 (92.6)
Neomycin	2 (2.9)	—	66 (97.1)
Norfloxacillin	59 (86.8)	—	9 (13.2)
Oxytetracycline	13 (19)	15 (22)	40 (58.8)
Streptomycin	3 (4.4)	—	65 (95.6)
Sulphonamides	7 (10.3)	21 (30.9)	40 (58.8)
Tetracycline	10 (14.7)	14 (20.6)	44 (64.7)
Trimethoprim	16 (23.5)	6 (8.8)	46 (67.6)
Total (%)	215 (28.7)	70 (9.4)	463 (61.9)

**Table 4:** Multidrug resistance patterns of *Escherichia coli* isolates.

Number of antimicrobial agents	Isolates from children (*n* = 68)	Number of isolates (%)
	Multidrug resistance patterns/number of isolates	
Two	SNM-OT (1); NOR-CLN (1); STM-NE (1)	3 (4.4)
Three	TE-NE-CHL (1); TRM-CLN-AMP (1); STM, CLN, OT (2)	4 (5.9)
Four	AMP-OT-NE-CLN (1); CHL, CLN, TRM, NE (1); CLN, TRM, TE, AMP (1)	3 (4.4)
Five	NOR-CLN-SNM-CHL-STM (1); OT-NE- -CHL-NE-TRM (1) TRM-NOR-CLN-SNM-TE (1); STM-NOR TE-CIP-CHL (1)	4 (5.9)
Six	NE-TE-CIP-CHL-TRM-NOR (2); NOR-CLN-SNM-OT AMP-STM (1); CHL-TRM-NOR-AMP-STM-TE (3).	6 (8.8)
Seven	TE-STM-CLN-NOR-CHL-NE-TRM (3); SNM-OT-CHL-TRM-NOR-STM-NE-TE (3); CLN-SNM-CHL-STM-NE-TE-AMP (2)	8 (11.8)
Eight	CIP-TRM-NOR-SNM-OT-AMP-STM-NE (2); TE-CIP-CHL-TRM-NOR-CLN-SNM-OT (3); AMP-STM-NE-TE-CIP-CHL-TRM-NOR (2);	7 (10.3)
Nine	OT-TE-AMP-STM-NE-CIP-CHL-TRM-CLN (2); NOR-AMP-CLN-SNM-OT-NE-CHL-TRM-STM (1); CHL-TRM-NOR-CLN-STM-NE-TE-SNM-OT (3)	6 (8.8)
Ten	TRM-AMP-STM-CLN-OT-NOR-CHL-NE-NOR-TE (1); TRM-CLN-SNM-CHL-STM-NE-CIP-TE-OT-NOR (1)	2 (2.9)
Total		43 (63.2%)

AMP-ampicillin; CLN-clindamycin; CHL-chloramphenicol; CIP-ciprofloxacillin; STM-Streptomycin; NE-neomycin; NOR-norfloxacillin; OT-oxytetracycline; TE-tetracycline; TRM-trimethoprim; SNM-sulphonamides; *n* = number.

**Table 5:** Antimicrobial resistance percentage of pathogenic *Escherichia coli* strains/virulence genes.

Antimicrobial agents	Detected virulence genes/strains in children (*N* = 38)													
	Resistant pathogenic *Escherichia coli* strains or genes/(%)													
	STEC (*n* = 10)		Atypical EPEC (only *eae*^+^) (*n* = 5)		EAEC *(eat* gene) (*n* = 14)		EHEC (*n* = 3)		DAEC *(dae* gene) (*n* = 2)		St (ETEC) (*n* = 4)		Total	
	R	S	R	S	R	S	R	S	R	S	R	S	R	S
Ampicillin	10(100)	—	5(100)	—	14 (100)	—	3(100)	—	2(100)	—	4(100)	—	38 (100)	—
Streptomycin	9(90)	1 (10)	4(80)	1 (20)	14 (100)	—	3(100)	—	2(100)	—	4(100)	—	36 (94.7)	2 (5.3)
Neomycin	10(100)	—	5(100)	—	14 (100)	—	3(100)	—	2(100)	—	4(100)	—	38 (100)	—
Tetracycline	8(80)	2 (20)	4(80)	1 (20)	12 (85.7)	2 (14.3)	2(66.7)	1(33.3)	2(100)	—	4(100)	—	32 (84.2)	6 (15.8)
Ciprofloxacillin	—	10 (100)	—	5 (100)	—	14 (100)	—	3(100)	—	2 (100)	2(50)	2 (50)	2 (5.3%)	36 (94.7)
Chloramphenicol	6(60)	4 (40)	3(60)	2 (40)	9 (64.3)	5 (35.7)	1(33.3)	2(66.7)	2(100)	2 (100)	3(75)	1 (25)	24 (63.2)	14 (36.8)
Trimethoprim	6(60)	4 (40)	4(80)	1 (20)	11 (78.6)	3 (21.4)	1(33.3)	2(66.7)	2(100)	—	4(100)	—	28 (73.7)	10 (26.3)
Norfloxacillin	3(30)	7 (70)	3(60)	2 (40)	8 (57.1)	6 (42.9)	—	3(100)	1(50)	1 (50)	3(75)	1 (25)	18 (47.4)	20 (52.6)
Clindamycin	10(100)	—	4(80)	1 (20)	13 (92.9)	1 (7.1)	3(100)	—	2(100)	—	4(100)	—	36 (94.7)	2 (5.3)
Sulphonamides	5(50)	5(50)	4(80)		12 (85.7)	2 (14.3)	2(66.7)	1(33.3)	2(100)	—	4(100)	—	29 (76.3)	9 (23.7)

*N* = number of strains; *R* = resistant; *S* = susceptible.

**Table 6:** Multidrug resistance of pathogenic *Escherichia coli* strains.

Number of drugs resisted	Multidrug resistance patterns/number of strains	Number of strains/%
Four	CLN-SNM-CHL-TRM (1); AMP-CHL-NE-STM (2)	3 (7.9)
Five	AMP-NE-CHL-NOR-SNM-(1); CLN-TRM-OT-STM-AMP (2); STM-NOR-CLN-SNM-TE (3)	6 (15.9)
Six	NE-OT-CHL-TRM-NOR-CLN (1); AMP-STM-NE-OT-CHL-TRM (1); NOR-CHL-NE-SNM-CLN-AMP-(2)	4 (10.5)
Seven	STM-CLN-NE-SNM-CHL-TRM-NOR (2); NE-NOR-CLN-SNM-OT-AMP-STM (1); CHL-TRM-NE-OT-STM-TE-CLN (1)	4 (10.5)
Eight	SNM-TRM-NOR-TE-CIP-OT-AMP-NE (4); STM-AMP-CHL-TRM-CLN-TE-NOR-OT (3); AMP-STM-NE-TE-CIP-CHL-CLN-TRM (3)	10 (26.4)
Nine	CLN-NOR-TRM-STM-NE-CIP-CHL-AMP-TE (2); NOR-AMP-CLN-SNM-OT-NE-CHL-TRM-STM (3); AMP-STM-NE-TE-SNM-OT-TE-CHL-CIP-TRM (3)	8 (21)
Ten	CLN-NE-CHL-AMP-OT-SNM-TRM-STM-NOR-TE (2) AMP-OT-CIP-TRM-TE-CHL-CLN-TRM-NE-SNM (1)	3 (7.9)
Total		38 (100%)

AMP-ampicillin; CLN-clindamycin; CHL-chloramphenicol; CIP-ciprofloxacillin; STM-streptomycin; NE-neomycin; NOR-norfloxacillin; OT-oxytetracycline; TE-tetracycline; TRM-trimethoprim; SNM-sulphonamides.

**Table 7:** Multidrug resistance index of pathogenic *Escherichia coli* strains.

(*n* = 38)	
MDR index value	Number of strains/%
0.3	—
0.4	3 (7.9)
0.45	6 (15.9)
0.54	3 (7.9)
0.6	4 (10.5)
0.7	10 (26.4)
0.8	8 (21)
0.9	4 (10.5)
Total	38 (100%)

(*n* = number of strains).

## Data Availability

The datasets used and analyzed throughout the study can be obtained from the corresponding author upon reasonable request.

## Abbreviations

A/E: Attaching and effacing
CFU: Colony forming unit
cGMP: Cyclic guanine monophosphate
CI: Confidence interval
COR: Crude odd ratio
DAEC: Diffusely adherent *Escherichia coli*
DDW: Double distilled water
DEC: Diarrheagenic *Escherichia coli*
DNA: Deoxyribonucleic acid
eae: *Escherichia coli* attaching and effacing gene
EDTA: Ethylene diamine tetra-acetic acid
EIEC: Enteroinvasive *Escherichia coli*
EPEC: Enteropathogenic *Escherichia coli*
EtBr: Ethidium bromide
ETEC: Enterotoxigenic *Escherichia coli*
EVC: *Escherichia coli* verocytotoxin
GTPase: Guanine triphosphatase
H: Flagellar antigen
IMViC: Indole methyl red voges proskauer citrate
LEE: Locus of enterocyte effacement
LT1: Heat labile toxin type one
M.a.s.l: Meter above sea level
MDR: Multidrug resistance
MDRI: Multidrug resistance index
MDa: Megadalton
MF: Method of feeding
NT: Navel treatment
O: Somatic antigen
*p*: Probability
PCR: Polymerase chain reaction
Rpm: Revolution per minute
SFT: Supplementary feed type
STEC: Shiga toxin-producing *Escherichia coli*
SIM: Sulfide indole motility
SPSS: Statistical procedure for social science
St: Heat stable toxin
Stx: Shiga toxin
Stx1 and Stx2: Shiga toxin gene-one and two
TBE: Tris base boric acid
TD: Types of diarrhea
TFCF: Time of first colostrum feeding
Tir: Translocated intimin receptor
TSI: Triple sugar iron
*χ* ^2^: Chi-square.
